# Identification of Therapeutic Targets for the Selective Killing of HBV-Positive Hepatocytes

**DOI:** 10.3390/jpm11070649

**Published:** 2021-07-10

**Authors:** Chien-Jung Huang, Lily Hui-Ching Wang, Yu-Chao Wang

**Affiliations:** 1 Institute of Biomedical Informatics, National Yang Ming Chiao Tung University, Taipei 112304, Taiwan; shana96300@gmail.com; 2 Institute of Molecular and Cellular Biology, National Tsing Hua University, Hsinchu 300044, Taiwan; lilywang@life.nthu.edu.tw; 3 Department of Medical Science, National Tsing Hua University, Hsinchu 300044, Taiwan

**Keywords:** hepatitis B virus, hepatocytes, conditionally essential genes, weighted co-expression network, random walk with restart, support vector machine, therapeutic targets

## Abstract

The hepatitis B virus (HBV) infection is a major risk factor for cirrhosis and hepatocellular carcinoma. Most infected individuals become lifelong carriers of HBV as the drugs currently used to treat the patients can only control the disease, thereby achieving functional cure (loss of the hepatitis B surface antigen) but not complete cure (elimination of infected hepatocytes). Therefore, we aimed to identify the target genes for the selective killing of HBV-positive hepatocytes to develop a novel therapy for the treatment of HBV infection. Our strategy was to recognize the conditionally essential genes that are essential for the survival of HBV-positive hepatocytes, but non-essential for the HBV-negative hepatocytes. Using microarray gene expression data curated from the Gene Expression Omnibus database and the known essential genes from the Online GEne Essentiality database, we used two approaches, comprising the random walk with restart algorithm and the support vector machine approach, to determine the potential targets for the selective killing of HBV-positive hepatocytes. The final candidate genes list obtained using these two approaches consisted of 36 target genes, which may be conditionally essential for the cell survival of HBV-positive hepatocytes; however, this requires further experimental validation. Therefore, the genes identified in this study can be used as potential drug targets to develop novel therapeutic strategies for the treatment of HBV, and may ultimately help in achieving the elusive goal of a complete cure for hepatitis B.

## 1. Introduction

Hepatitis B virus (HBV) is a double-stranded DNA virus and a member of the family *Hepadnaviridae*. HBV infection causes hepatitis B, which is an infectious disease that leads to acute or chronic hepatitis. According to a report by the World Health Organization in 2015, an estimated 257 million people were living with chronic HBV infection globally, and the prevalence in adults in the sub-Saharan regions and East Asia was 5–10% [1,2]. In the case of HBV carriers, the HBV antigens, mainly hepatitis B surface antigen (HBsAg), can be detected in the blood of the patients. Based on the transmission of HBV, this infection can be divided into blood and body fluid infections, and the mode of infections can further be divided into vertical and horizontal infections. Vertical infection refers to the transfer of the infection from the HBV carrier mothers to their newborn babies via the placenta or birth canal, whereas horizontal infection refers to the infection caused by contact with the blood or body fluid of the carriers, for example, blood transfusion, injection, and the tattooing process. As vertical infection is the main source of infection, Taiwan has been administering the hepatitis B vaccine since 1984. The vaccination program has effectively blocked the vertical infection of hepatitis B, and the prevalence of hepatitis B among those younger than 20 years old decreased from 9.8% in 1984 to 0.6% in 2004 [3]. However, the carrier rate of hepatitis B in adults is approximately 15% in Taiwan. Even if the hepatitis B vaccine is effective, most infected individuals will become lifelong carriers of HBV as there is no cure for HBV infection. Therefore, controlling or even curing the HBV infection is an essential public health issue that must be addressed. At present, there are two types of agents available for the treatment of HBV infection: interferons and nucleotide analogs. Interferons have antiviral, anti-proliferative, and immunomodulatory effects, whereas nucleotide analogs suppress the replication of HBV [4]. Nevertheless, the current drugs for treating HBV infection can only control the disease and achieve a functional cure (loss of HBsAg). In addition, regardless of the type of drug, drug resistance is eventually seen in the patients [5,6]. For HBV carriers, the virus always exists in the liver and the HBV DNA is permanently integrated into the host genome [7]. Although the virus may not harm the liver directly, the human immune cells will attack the liver cells when they recognize the virus in the liver cells, resulting in inflammation and impairment of liver functions, which may eventually lead to cirrhosis and hepatocellular carcinoma (HCC). Therefore, how to achieve complete cure (elimination of infected hepatocytes and the cells with integrated HBV DNA) for HBV carriers is still a problem to be solved [8]. In order to eliminate HBV-infected hepatocytes, our strategy was to identify the target genes that could be used for the selective killing of HBV-positive hepatocytes. The target genes must be crucial for survival of the HBV-positive hepatocytes but insignificant for the survival of HBV-negative hepatocytes.

Genes that are critical for the survival of organisms are called essential genes. However, the essentiality of a gene is not an intrinsic property, indicating that the gene may be essential in some conditions but not essential in other circumstances. Essentiality is highly dependent on a variety of factors, such as the function and expression pattern of the gene, genetic context of the organism, and environment. [9] This property makes essential genes therapeutic targets of diseases. In the past, the method of identifying essential genes was to observe the survival of the cells with target gene knock-out or knock-down through experiments. However, different experimental methods may produce different results. For example, the CRISPR-based methods identify more essential genes than the siRNA-based methods [10]. Genes with different essentialities in different situations are called conditionally essential genes (CEGs) [11,12], which can be applied to develop more effective or more specific drugs. In cancer therapies, CEGs are essential for specific tumor cells but not for other normal cells [13]. For example, poly (ADP-ribose) polymerase (PARP) inhibitors have been approved for the treatment of ovarian cancer. As the PARP protein is only required for the *BRCA1*- or *BRCA2*-deficient cells, it can be used for selectively eliminating the cancer cells [14]. The concept of this treatment, which takes advantage of the different essentialities of different cells, is similar to our strategy to identify therapeutic targets for the selective killing of HBV-positive hepatocytes.

With advancements in technology, many high-throughput technologies have been developed in recent years to explore biomedicine, and many datasets have also been deposited in public databases. In this study, we collected the gene expression profiles of hepatitis B from a public database and developed a computational method to identify the CEGs in the context of HBV infection. The random walk with restart (RWR) algorithm and the machine learning approach with support vector machine (SVM) were used for the identification of CEGs. The identified CEGs were found to play key roles in the survival of the HBV-infected liver cells but were not related to the survival of the normal liver cells. Therefore, these CEGs can be used as therapeutic targets to design potent drugs for HBV infection, which can selectively kill the HBV-infected liver cells without affecting the normal liver cells. Moreover, by applying the method for the regeneration of normal liver cells, we hope to achieve the ultimate goal of a complete cure for hepatitis B.

## 2. Materials and Methods

### 2.1. Overview of the Method

**Figure 1:**
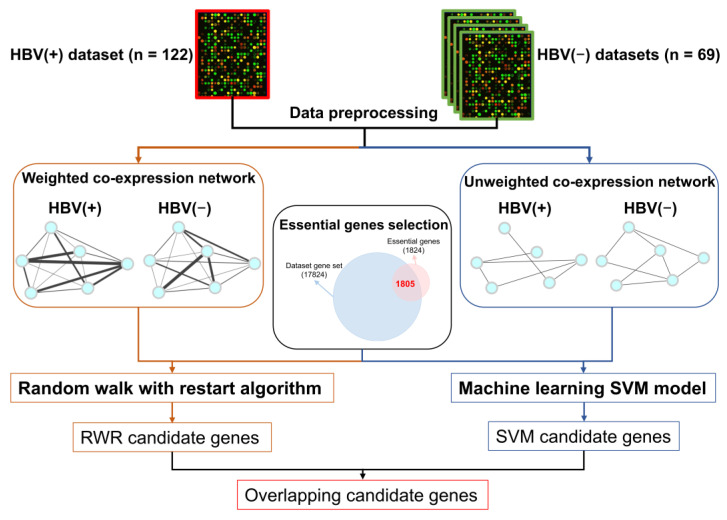
Schematic representation of the workflow of this study.

The workflow of this study is shown in Figure 1. The gene expression data of HBV-positive (HBV(+)) and HBV-negative (HBV(−)) samples were collected and downloaded from a public database. After data preprocessing, the two types of networks, weighted co-expression networks and unweighted co-expression networks, were constructed. With the selected essential genes, two algorithms were used to identify the CEGs: the RWR algorithm and the SVM approach. The identified CEGs were the intersection of the candidate genes predicted by the two algorithms.

### 2.2. Datasets and Sample Characteristics

We curated the microarray gene expression data of the HBV(+) and HBV(−) samples from the Gene Expression Omnibus (GEO) database (GPL570 platform). Among the curated datasets, HBV(+) samples (*n* = 122) are chronic hepatitis B liver samples obtained from GSE83148 [15] and HBV(−) samples were obtained from GSE83148 (*n* = 6) [15], GSE6764 (*n* = 10) [16], GSE14668 (*n* = 11) [17], GSE38941 (*n* = 10) [18], GSE23343 (*n* = 7) [19], GSE28619 (*n* = 7) [20], GSE62029 (*n* = 10) [21], and GSE101685 (*n* = 8). None of the HBV(−) samples showed evidence of infection with hepatitis B, C, and HCC. Information about these curated datasets is presented in Table 1.

### 2.3. Data Preprocessing

Due to the different methods for the normalization of these curated datasets, we downloaded the raw data (CEL file) of each sample for merging and used the Robust Multichip Average (RMA) algorithm [22] (R package–affy_1.68.0) for normalization. Then, the batch effects were removed with the “removeBatchEffect” function (R package–limma_3.46.0) [23]. As the curated datasets contained expression profiles for protein-coding genes and non-coding RNAs, 17,824 protein-coding genes were selected for further analysis.

### 2.4. Selection of the Known Essential Genes

Based on the objectives of this study, the essential genes were selected as the seed nodes of the RWR algorithm. We downloaded the Homo sapiens essential genes annotation file from the Online GEne Essentiality (OGEE) database (version 2), which lists the essentiality consensus of 21,556 genes that are experimentally verified. A total of 7168 genes were labeled as “Essential” (marked as essential in all studies) or “Conditional” (marked as essential in some studies and non-essential in other studies). However, because each gene was labeled with a different number of studies, the genes with at least four studies (and three out of four) marked as essential were selected as the known essential genes in this study. Based on this criterion, a total of 1805 known essential genes were selected for further analysis.

### 2.5. RWR Algorithm

To execute the RWR algorithm, weighted co-expression networks for both HBV(+) and HBV(−) samples were constructed, respectively. First, the gene–gene Spearman correlation matrix was constructed by calculating the Spearman correlation coefficients between every gene pair. Then, the soft threshold picking method based on weighted gene co-expression network analysis (WGCNA) [24] was used to select the suitable power *β* for both HBV(+) and HBV(−) matrices. The value of power *β* was selected according to the scale-free topology criterion, which aims to simulate the natural biological network structure [25]. The steps for constructing weighted gene co-expression networks are shown in Supplementary Figure S1.

RWR is a ranking algorithm that simulates a random walker starting from the seed nodes, which represent the known targets with specific properties, thereby calculating the probability of each node as a novel target. It has been applied to networks of different structures (e.g., microRNA-target gene network [26] and protein–protein interaction network [27]) and has been successfully used to predict potential disease-associated microRNAs/genes. The RWR formula is as follows:

(1) $$P_{t+1}=\left(1-r\right)W^{T}P_{t}+rP_{0}$$

where *W* is the row-normalized transition matrix, which can be converted from our network correlation matrix given that power *β* is selected, *W_ij_* of the transition matrix *W* is the transition probability from node *i* to node *j*, $P_{0}$ is the *N ×* 1 initial score vector (*N* is the number of genes in the networks), in which the seed nodes are assigned equal values and the sum of the values equals 1, where other non-seed nodes are assigned 0, $P_{t}$ is the score vector after *t* iterations, and *r* is the restart probability of the walker returning to the seed nodes, which can ensure the importance of the seed nodes. The implication of the restart probability is that in every step, scores with *r* ratio remained in the seed nodes, whereas others with 1 − *r* ratio were shared with neighbors. After *t* iterations of this algorithm, the loop stopped when $\|P_{t+1}-P_{t}\|<10^{-6}$ [28], indicating that the score of all of the nodes reached a steady state. In this case, $P_{t+1}$ was the final score vector, where nodes (genes) with higher scores were considered more likely to be the seed nodes. With known essential genes as the seed nodes, the scores determined by RWR were defined as the essential scores for each gene. The steps for calculating the essential difference scores are shown in Supplementary Figure S2. Genes with larger essential difference scores are more likely to be the CEGs in HBV-infected hepatocytes. To avoid false positives in the RWR results, we also performed a permutation test to calculate an empirical *p*-value for each gene [27,29,30,31].

Based on the essential difference score and the corresponding empirical *p*-values calculated for each gene, three criteria were used to screen RWR candidate genes: (1) genes with empirical *p*-values less than 0.05, (2) genes with the top 10% essential difference score, and (3) genes with the top 25% essential score ranking in the HBV(+) network and the bottom 25% essential score ranking in the HBV(−) network. Genes that met all three criteria were identified as RWR candidate genes.

### 2.6. SVM Approach

In addition to the RWR algorithm, a machine learning method using SVM was also used for the identification of CEGs. Machine learning is one of the main methods for predicting essential genes [32,33]. We referred to the study by Hwang et al. to predict essential genes using network features and SVM [34]. As some network features can only be computed in unweighted networks, unweighted gene co-expression networks for both HBV(+) and HBV(−) samples were constructed, respectively. Similarly, correlation matrices for both conditions were first constructed. Subsequently, the concept of choosing a hard threshold according to the scale-free topology criterion was employed to transform the weighted networks into unweighted gene co-expression networks. That is, we set the cut-off in the range [0.1–0.9] in increments of 0.1, and tested whether the resulting networks of each cut-off displayed a scale-free topological structure.

Seven network features were calculated to train the SVM classification model: degree (K), betweenness centrality (BC), closeness centrality (CC), clustering coefficient (CCo), neighbors’ intra-degree (NID), essentiality index (EI), and common-function degree (CFK) [34]. Among these features, K, BC, CC, CCo, and NID were calculated using python package—NetwrokX_1.11 [35], EI was calculated using the known essential genes we filtered, and CFK was calculated using the Gene Ontology (GO) annotation file downloaded from the National Center for Biotechnology Information (NCBI) website. The seven network features for each gene were calculated in HBV(+) and HBV(−) unweighted gene co-expression networks, respectively. The steps for calculating the essential difference probabilities are shown in Supplementary Figure S3. Genes with larger essential difference probabilities are more likely to be the CEGs in HBV-infected hepatocytes.

For the selection of SVM candidate genes, because there is no empirical *p*-value for each gene, the following two criteria as in RWR were used: (1) genes with the top 10% difference essential probability, and (2) genes with the top 25% essential probability ranking in the HBV(+) network and the bottom 25% essential probability ranking in the HBV(−) network.

### 2.7. Gene Set Enrichment Preranked Analysis (GSEAPreranked)

Based on the ranked essential difference scores from RWR and the ranked essential difference probabilities from SVM, we obtained the average ranking of each gene in the networks. Given the ranked list of genes, the GSEAPreranked analysis for enriched pathways and functions was performed with the “gseKEGG” and “gseGO” function (R package—clusterProfiler_3.18.1) [36]. The enriched pathways and functions were analyzed with the Kyoto Encyclopedia of Genes and Genomes (KEGG) (release: 10 June 2021) and GO biological process (GOBP) gene sets (release: 1 February 2021) embedded in clusterProfiler. Those gene sets with Benjamini–Hochberg adjusted *p*-values less than 0.05 were selected as the enriched pathways/functions. The “simplify” function in clusterProfiler was further employed to remove redundancy of enriched GO terms. The enrichment map showing the correlation between enriched GO terms was displayed using the “emapplot” function (R package—enrichplot_1.13.0.993).

## 3. Results

### 3.1. Data Preparation

Using the microarray gene expression profiles for HBV(+) and HBV(−) samples downloaded from the GEO database, a computational framework was developed to predict the CEGs. After normalization of the raw data of each sample, the batch effect was removed, and 17,824 protein-coding genes were selected for further analysis. The gene expression profile distributions for all samples are shown in Supplementary Figure S4.

The known essential genes were curated before proceeding with the RWR and SVM analyses. The Homo sapiens essential genes annotation file was downloaded from the OGEE database, which labeled 7168 genes as essential genes. In addition, we also investigated the number of known human essential genes and found that only ~10% of ~20,000 genes in human cells are essential for cell survival [13,37]. Therefore, 7168 essential genes were further screened. Based on the data provided by OGEE, each gene was marked with different numbers of essentiality status—Essential (E) or Non-Essential (NE)—according to the experimental results of each study (dataset). In other words, a gene can be essential in some datasets but non-essential in other datasets. The distribution of marked datasets for 7168 essential genes indicated that most of the essential genes were marked in 8 datasets. The genes that were marked as ‘E’ in at least four datasets or marked as ‘E’ in three out of four datasets were selected as known essential genes with high confidence. Finally, 1805 known essential genes were selected for further analysis.

### 3.2. Using RWR to Identify the CEGs

For the RWR analysis, the weighted gene co-expression networks for HBV(+) and HBV(−) samples were constructed, where the power *β* needed to be determined first. The different power *β*’s and their corresponding $R^{2}$ in the scale-free topological model fitting are shown in Supplementary Figure S5. The results showed that $R^{2}$ increased as the power *β* increased. Based on the results shown in Supplementary Figure S5 and the criteria that $R^{2}\geq 0.7$, power *β* was selected as six for HBV(+) and four for HBV(−) weighted networks, and the corresponding $R^{2}$ was 0.71 and 0.74 in HBV(+) and HBV(−), respectively.

The essential score for each gene in the HBV(+) and HBV(−) weighted gene co-expression networks was determined using the RWR algorithm, respectively. The quantile–quantile plot showed that there was only a small deviation in the essential score distribution of genes in HBV(+) and HBV(−) networks (Supplementary Figure S6A), demonstrating that the essential scores for HBV(+) and HBV(−) were comparable. The essential difference score was calculated by subtracting the essential score of HBV(−) from the essential score of HBV(+), and the distribution of essential difference scores is shown in Supplementary Figure S6B. Genes with larger essential scores indicated that they are more likely to be essential genes. Therefore, the genes with larger essential difference scores indicate that they are more likely to be essential in HBV(+) samples but less likely to be essential in HBV(−) samples, making them CEGs. Based on the three criteria mentioned above, that is, essential difference score ranking, essential score ranking, and empirical *p*-value, 309 candidate genes were identified using the RWR algorithm.

Referring to the literature [38,39], the restart probability *r* in Equation (1) was initially set as 0.7, and 309 candidate genes were identified. Subsequently, we tested the impact of different *r* values on the identified RWR candidate genes. As the *r* value used in the RWR algorithm were between 0 and 1, we set the *r* value in the range [0.1–0.9] in increments of 0.2, and tested whether the identified RWR candidate genes of each *r* value were significantly different. The number of candidate genes identified for different values of *r* is listed in Supplementary Table S1. The number of overlapped candidate genes and their Jaccard similarities are listed in Supplementary Table S2. The average Jaccard similarity for *r* = 0.7 is 0.72, demonstrating that the *r* value has little impact on our final RWR candidate genes.

### 3.3. Using SVM to Identify the CEGs

For the SVM analysis, seven network topological features that could be used to predict essential genes were calculated. As only four features could be calculated in the unweighted network, the unweighted networks of HBV(+) and HBV(−) samples were constructed first. Similarly, the Spearman correlation coefficients for each gene pair were calculated, leading to correlation matrices for both conditions. Subsequently, the hard threshold was determined. Based on the preset cut-offs and the corresponding scale-free model fitting index $R^{2}$ shown in Supplementary Figure S7, the hard threshold was selected as 0.5 for both HBV(+) and HBV(−), such that the corresponding $R^{2}\geq 0.7$. The scale-free model fitting index $R^{2}$ and the identified *γ* (slope parameter when fitting power law distribution) of each cut-off, as well as the degree statistical characteristics of the corresponding unweighted network, are listed in Supplementary Table S3. The results showed that in the HBV(+) network, when the cut-off was 0.1–0.2, it did not conform to the power law (the slope parameter should be positive), even with a very high $R^{2}$. Therefore, we did not choose 0.1 and 0.2 as the hard threshold for unweighted network construction. With 0.5 as the hard threshold, the weighted HBV(+) and HBV(−) networks can be transformed into unweighted networks. During the transformation, some genes had a degree of zero in the unweighted network and were excluded from further analysis, leading to 16,419 genes remaining for SVM analysis.

Based on the unweighted HBV(+) and HBV(−) networks, the SVM classification model was used to predict candidate genes. As SVM is a supervised method, both positive and negative data are required for model training. The positive group of training data consisted of the 1754 known essential genes (after mapping 1805 known essential genes to the unweighted networks, the number of known essential genes in the unweighted networks was 1754). However, based on the principle of the essentiality of genes, it is difficult to define non-essential genes. We referred to the method described by Yang et al., which used random sampling for non-essential gene selection [32]. In this study, the negative group of training data was 1754 genes that were randomly selected from the 14,665 genes other than the known essential genes, and the prediction data included 14,665 remaining genes (Supplementary Figure S3). The non-essential genes were randomly selected 1000 times, and thus, 1000 SVM models were trained to provide essential status for each gene in the network. After 1000 SVM classifications, the essential probability, which was calculated by dividing essential counts (the number of times to be predicted as essential genes) by 1000, was obtained for each gene in HBV(+) and HBV(−) networks, respectively. Furthermore, the essential difference probability was calculated by subtracting the essential probability of HBV(−) from the essential probability of HBV(+). Based on the two criteria mentioned above, that is, essential difference probability ranking and essential probability ranking, 688 candidate genes were identified using the SVM algorithm.

Cross-validation was performed to verify the prediction rate of this model. The 1754 known essential genes and 1754 randomly selected non-essential genes were divided into 70% training data and 30% testing data to perform cross-validation. The procedure was repeated 1000 times and the average precision, recall, and accuracy were calculated. The three indicators are between 0.70 and 0.72 in HBV(+) and HBV(−), indicating that this SVM model can be used to predict essential genes.

Although the network topological features were demonstrated to be useful for predicting essential genes, we wanted to investigate whether they can be used for prediction in both HBV(+) and HBV(−) networks. Based on the SVM model construction method, the essential genes were fixed, and non-essential genes were randomly selected. The Mann–Whitney U test was used to investigate whether these seven network features were significantly different between essential and non-essential genes. The results of statistical tests are listed in Supplementary Table S4, indicating that regardless of the HBV(+) or HBV(−) unweighted networks, essential and non-essential genes have significant differences in all seven network features, verifying that these features can be directly applied to our networks.

### 3.4. GSEAPreranked for the Average Ranking Gene List

**Figure 2:**
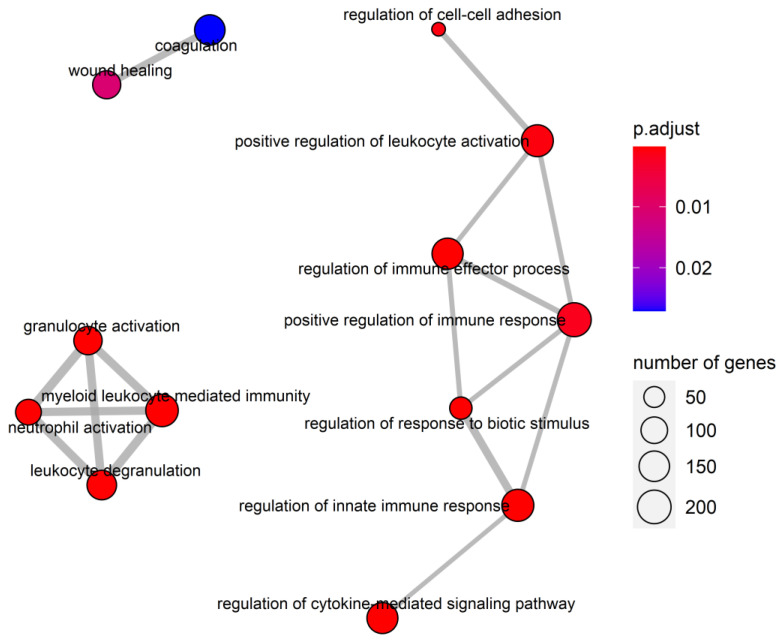
The enrichment map of the gene set enrichment analysis (GSEA) of Gene Ontology (GO) gene sets. The edges between the enriched GO terms indicate that the Jaccard similarity among the gene sets was larger than 0.25, in which the width represents the level of Jaccard similarity. The size of nodes shows the size of GOBP gene sets. The color of nodes indicates the level of adjusted *p*-values for enriched gene sets.

Given the ranked gene lists based on essential difference scores from RWR and essential difference probabilities from SVM, GSEAPreranked analysis was performed to determine whether some pathways showed significant enrichment. The given ranked gene list was positively enriched in 26 KEGG pathways and 33 GOBP functions (Supplementary Tables S5 and S6). It is worth noting that one of the enriched KEGG gene sets was Fc gamma R-mediated phagocytosis (adjusted *p*-value = 0.047). It has been reported that Fc gamma receptors are involved in the emerging immunotherapy of chronic HBV infection treatment, which can durably suppress the levels of HBsAg and HBV DNA via Fcγ receptor-dependent phagocytosis [40,41]. In addition, pathways related to cell survival such as apoptosis (adjusted *p*-value = 0.032) have also been identified, suggesting that the designed workflow may be used to identify CEGs. We calculated the Jaccard coefficient of enriched GOBP gene sets and further analyzed the correlation between the results. The threshold of the Jaccard coefficient was set to 0.25, and the nodes with degree less than one were filtered. The enrichment map showed that the enriched GO functions can be divided into three groups (Figure 2). The main group is related to the regulation of immune responses, including cell–cell adhesion, leukocyte activation, immune effector process, immune response, response to biotic stimulus, innate immune response, and cytokine-mediated signaling pathway, indicating that most of the enriched GOBP gene sets are related to immune regulation. The second group is related to the leukocyte, including granulocytes activation, myeloid leukocyte-mediated immunity, neutrophil activation, and leukocyte degranulation. The last group includes coagulation and wound healing.

### 3.5. Candidate Genes Analysis

**Figure 3:**
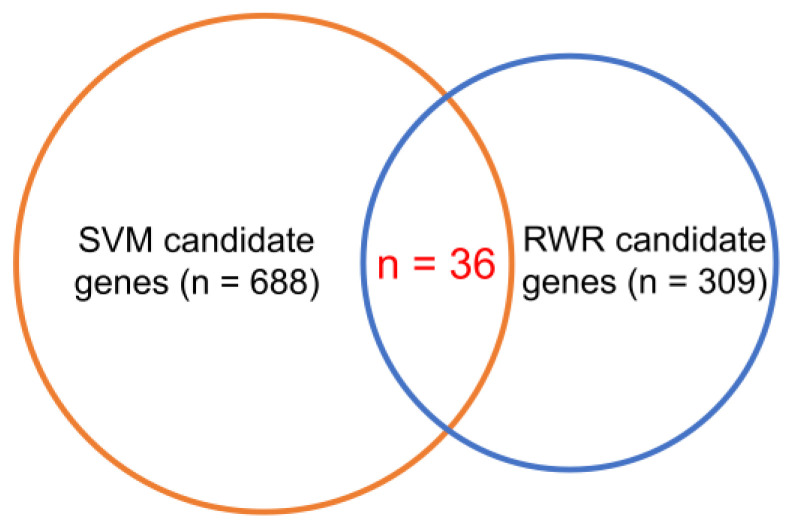
The 36 overlapping candidate genes are the intersection of the random walk with restart (RWR) and support vector machine (SVM) candidate genes.

With 309 RWR and 688 SVM candidate genes identified, these two gene sets were intersected, resulting in 36 overlapping candidate genes, which are potentially the CEGs (Figure 3). The ranked gene list of these 36 overlapping candidate genes is listed in Table 2, where the genes with higher ranking were more likely to be CEGs in HBV-infected hepatocytes, according to our analyses.

**Figure 4:**
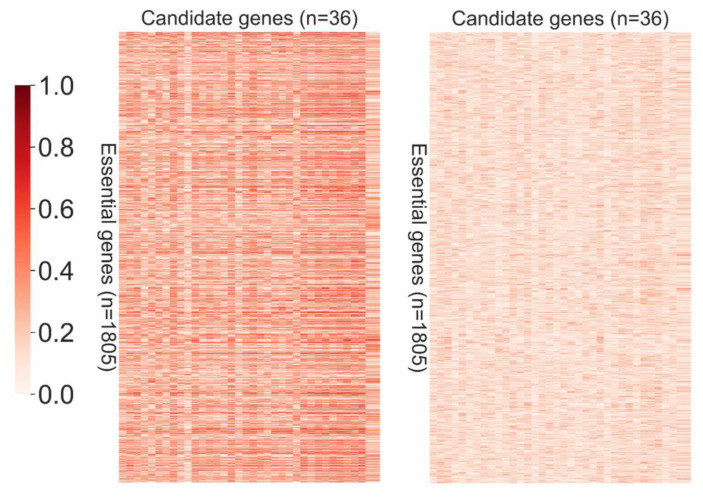
Correlation between the overlapping candidate genes and known essential genes. Left: HBV(+) samples, right: HBV(−) samples.

To validate the overlapping candidate genes, we first investigated whether the gene expression of these identified genes was more significantly correlated with known essential genes in HBV(+) samples as compared to in HBV(−) samples. The Spearman correlation coefficients of gene expression between 36 overlapping candidate genes and 1805 known essential genes were calculated, and the correlation heatmaps of HBV(+) and HBV(−) are shown in Figure 4. The spearman correlation coefficients are taken as absolute values, which were between 0 and 1, so the darkest color in the color bar showed the highest correlation. The results show that the correlation between the identified overlapping candidate genes and known essential genes is higher in HBV(+) samples, but lower in HBV(−) samples, which means that the 36 overlapping candidate genes are more likely to be essential genes in HBV(+) samples, but not in HBV(−) samples.

**Figure 5:**
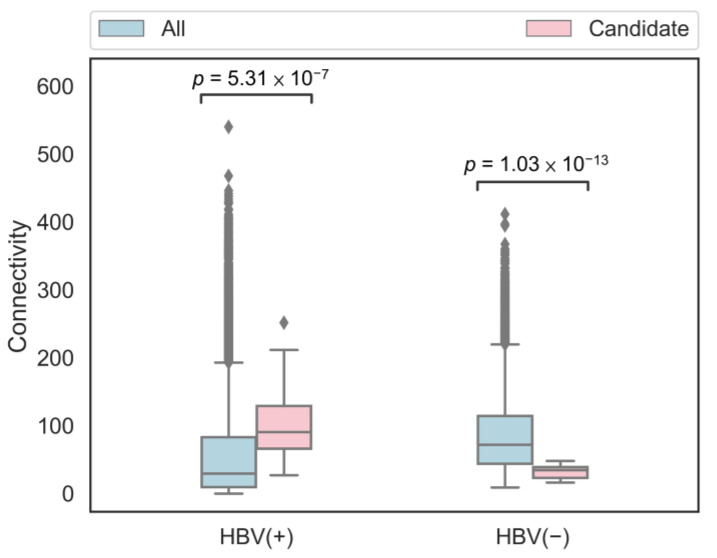
The boxplots of connectivity for all/candidate genes in the HBV(+) and HBV(−) weighted networks. *p*-value was obtained using the Mann–Whitney U test.

Many studies have indicated that essential genes are in the network center (that is, the connection with other genes was higher), but non-essential genes were the opposite [13,61]. Therefore, the connectivity of the overlapping candidate genes in both HBV(+) and HBV(−) networks was analyzed. The boxplots of the connectivity for all genes and 36 overlapping candidate genes in the HBV(+) and HBV(−) weighted networks are shown in Figure 5. Connectivity is a connection degree indicator for genes and other genes. The connectivity of these overlapping candidate genes is relatively large in the HBV(+) network, whereas the connectivity of the overlapping candidate genes in the HBV(−) network is relatively small. According to the literature, these 36 overlapping candidate genes are more likely to be essential genes in HBV(+) samples, but are less likely in HBV(−) samples, which are the CEGs we would like to identify.

## 4. Discussion

The aim of this study was to identify the CEGs among HBV(+) and HBV(−) samples, which can be used as therapeutic targets for treating HBV infection. For the purpose of this study, HBV(+) and HBV(−) gene co-expression networks were constructed. In the first step of network construction, the GSE83148 dataset was downloaded from the GEO database, which contains 122 HBV(+) and 6 HBV(−) samples, and the Spearman correlation coefficients for every gene pair were calculated. As the calculation of Spearman correlation coefficients is limited by the sample size, only six HBV(−) samples could have caused deviations in the calculation of correlation. Therefore, we further collected all available HBV(−) samples (*n* = 63) from the GPL570 platform (the same as the GSE83148 dataset) and demonstrated that these samples were not infected with hepatitis B, C, and HCC. As these HBV(−) samples were retrieved from different datasets and different normalization methods were applied, the CEL file of raw data for each sample was downloaded, and RMA normalization was performed for all HBV(+) and HBV(−) samples. In addition, batch effects were removed. Even with all the HBV(−) samples in the GPL570 platform, the sample sizes among 122 HBV(+) and 69 HBV(−) samples were still not balanced. Consequently, there may have been some deviations in calculating the correlations and comparing the two networks. We believe that if more HBV(−) samples can be collected in the future, the identified CEGs can be confidently identified. Moreover, HBV(−) gene expression profiles were retrieved from samples from different countries, which may have different genetic backgrounds (Table 1). Since different genetic backgrounds of the HBV(+) and HBV(−) samples may influence the results, further studies are needed to investigate the possible impact of various genotypes on the identification of CEGs.

Based on two different approaches, RWR and SVM, 36 overlapping candidate genes were identified in this study. We further searched the literature for the 36 candidate genes to investigate their associations with HBV infection. Several genes have been reported to be associated with HBV replication, integration, and other related functions. The *APOBEC3* family has antiviral activity against retroviruses and can also inhibit HBV replication [42,43]. *UBE2H* was a breakpoint of HBV integration in plasma DNA for HBV-related HCC samples, and was upregulated in HCC [50,51]. *TCF7L2* may participate in the upregulation of HBV core promoter activity via the interaction with the enhancer region and *PUF60* [52]. Multiple TRIM proteins with E3 ligase function can inhibit HBV transcription [55]. *ARHGEF12* was recurrently inserted by HBV in tumor-adjacent tissues of HBV-related HCC samples to increased its gene expression [57]. HBV could have a large effect on the concurrent DNA methylation of *CDH1*, *DNMT3b,* and *ESR1* in the serum of HBV-related HCC samples [58]. *ARRB1* has been indicated to promote HCC, and was upregulated by hepatitis B virus X protein (HBx) in mouse models [59]. Interestingly, among other candidate genes, some genes are related to the progression of HCC, including, *GATAD1* [44,45], *FOXK1* [46], *RALGAPA2* [47], *PDE4A* [48], *TRIP6* [49], *SPTBN1* [53], *PTP4A3* [54], *TRIM4* [56], and *LMNB1* [60]. These results suggest that the 36 candidate genes are associated with the functions of HBV and hepatocytes; however, this association needs to be further validated. Moreover, to investigate whether some enriched GOBP functions were shared among the 36 overlapping candidate genes, 273 RWR-specific genes, and 652 SVM-specific genes, functional annotation clustering was applied to identify the enriched GOBP functions. After removing redundancy, 37, 39, and 77 enriched GOBP functions were identified for overlapping genes, RWR-specific genes, and SVM-specific genes, respectively (Supplementary Figure S8). Only two GOBP functions were found to be shared among overlapping candidate genes and SVM-specific genes. Since RWR and SVM use different principles for identifying the CEGs, the result is not surprising. In addition, through literature surveys, we found that most of the 36 overlapping genes are related to HBV or hepatocytes (Table 2), demonstrating that application of these two methods rather than a single one can avoid the false positives. Therefore, the CEGs identified by both methods are more robust.

## 5. Conclusions

In contrast to conventional studies, we used the concept of CEGs to develop targeted drugs for the HBV-infected liver cells. We also developed a computational framework to predict the conditionally essential genes in the HBV-infected liver cells. This computational framework contains two algorithms: RWR and SVM. A total of 309 RWR and 688 SVM candidate genes were identified in this study. The RWR and SVM candidate genes were then intersected to identify the 36 overlapping candidate genes, which may be CEGs. The gene expression correlation heatmap of these 36 genes and 1805 known essential genes showed a higher similarity in the HBV(+) samples than the HBV(−) samples. The results of the connectivity analysis were consistent with the network characteristics of essential and non-essential genes mentioned in the literature. Essential genes exhibit higher connectivity, but non-essential genes do not. Most candidate genes are associated with the functions of HBV and hepatocytes, indicating that these 36 target genes could be used as potential drug targets to develop novel strategies for managing HBV infection and may ultimately help in achieving the elusive goal of a complete cure for hepatitis B.

## Figures and Tables

**Table 1:** The information of GEO microarray datasets used in the study.

Type	GEO ID	Sample Number	Country	Year
HBV(+)	GSE83148	122	China	2017
HBV(−)	GSE83148	6	China	2017
	GSE6764	10	USA	2007
	GSE14668	11	USA	2010
	GSE38941	10	USA	2012
	GSE23343	7	Japan	2010
	GSE28619	7	Spain	2012
	GSE62029	10	Italy	2015
	GSE101685	8	Taiwan	2019

**Table 2:** The ranked gene list of potential conditionally essential genes.

Ranking	Entrez ID	Gene Symbol	Association with HBV/HCC	Ranking	Entrez ID	Gene Symbol	Association with HBV/HCC
1	200030	*NBPF11*		19	27350	*APOBEC3C*	HBV [42,43]
2	284565	*NBPF15*		20	57798	*GATAD1*	HCC [44,45]
3	221937	*FOXK1*	HCC [46]	21	342979	*PALM3*	
4	148266	*ZNF569*		22	4335	*MNT*	
5	9883	*POM121*		23	57186	*RALGAPA2*	HCC [47]
6	2077	*ERF*		24	9743	*ARHGAP32*	
7	5141	*PDE4A*	HBV and HCC [48]	25	7205	*TRIP6*	HCC [49]
8	7328	*UBE2H*	HBV and HCC [50,51]	26	51479	*ANKFY1*	
9	84433	*CARD11*		27	6934	*TCF7L2*	HBV [52]
10	6711	*SPTBN1*	HCC [53]	28	7025	*NR2F1*	
11	11156	*PTP4A3*	HCC [54]	29	79719	*AAGAB*	
12	89122	*TRIM4*	HCC [55,56]	30	23365	*ARHGEF12*	HBV and HCC [57]
13	999	*CDH1*	HBV and HCC [58]	31	5269	*SERPINB6*	
14	23568	*ARL2BP*		32	56935	*SMCO4*	
15	60401	*EDA2R*		33	408	*ARRB1*	HBV and HCC [59]
16	7559	*ZNF12*		34	90268	*OTULIN*	
17	6310	*ATXN1*		35	81030	*ZBP1*	
18	4001	*LMNB1*	HCC [60]	36	342371	*ATXN1L*	

## Data Availability

Publicly available datasets were analyzed in this study. This data can be found here: https://www.ncbi.nlm.nih.gov/geo/. Accession numbers were shown in Materials and Methods. Data was accessed on 3 June 2021.

## Supplementary Materials

The following are available online at https://www.mdpi.com/article/10.3390/jpm11070649/s1, Figure S1: Steps for the construction of the weighted gene co-expression networks; Figure S2: Steps for calculating the essential difference score using the random walk with restart (RWR) algorithm; Figure S3: Steps for calculating the essential difference probability using the support vector machine (SVM) approach; Figure S4: The gene expression profile distributions across HBV(+) and HBV(−) samples after normalization; Figure S5: Different power *β*’s and their corresponding $R^{2}$ in the scale-free topological model fitting for the soft threshold selection; Figure S6: Distributions of the essential scores and essential difference scores; Figure S7: Different cut-offs and their corresponding $R^{2}$ in the scale-free topological model fitting for the hard threshold selection; Figure S8: The Venn diagram showing the relation between the enriched GOBP functions for overlapping candidate genes, RWR-specific genes, and SVM-specific genes. Table S1: The number of identified candidate genes for different values of *r*; Table S2: The number of overlapped candidate genes and their Jaccard similarities; Table S3: The scale-free model fitting index *R*^2^, the identified *γ*, and the degree statistical characteristics of the corresponding unweighted network for each cut-off; Table S4: Statistical test results comparing the network features among essential and non-essential genes; Table S5: The enrichment results of the enriched Kyoto Encyclopedia of Genes and Genomes (KEGG) gene sets based on the gene set enrichment analysis (GSEA); Table S6: The enrichment results of the enriched Gene Ontology (GO) gene sets based on the gene set enrichment analysis (GSEA).
