# Supplementary material for: Identification of Therapeutic Targets for the Selective Killing of HBV-Positive Hepatocytes
Source: J Pers Med. 2021 Jul 10;11(7):649. doi: 10.3390/jpm11070649 (PMC8307716; doi:10.3390/jpm11070649)
Supplement: Supplementary file 1 [file jpm-11-00649-s001.zip › jpm-1283494-supplementary.pdf]

Identification of therapeutic targets for the selective killing of HBV-positive hepatocytes

Chien-Jung Huang, Lily Hui-Ching Wang, and Yu-Chao Wang

Supplementary Materials

Supplementary Figures

Figure S1. Steps for the construction of the weighted gene co-expression networks.

Figure S2. Steps for calculating the essential difference score using the random walk with restart (RWR) algorithm.

Figure S3. Steps for calculating the essential difference probability using the support vector machine (SVM) approach.

Figure S4. The gene expression profile distributions across HBV(+) and HBV(−) samples after normalization.

Figure S5. Different power β's and their corresponding R^2 in the scale-free topological model fitting for the soft threshold selection.

Figure S6. Distributions of the essential scores and essential difference scores.

Figure S7. Different cut-offs and their corresponding R^2 in the scale-free topological model fitting for the hard threshold selection.

Figure S8: The Venn diagram showing the relation between the enriched GOBP functions for overlapping candidate genes, RWR-specific genes, and SVM-specific genes.

Supplementary Tables

Table S1. The number of identified candidate genes for different values of r.

Table S2. The number of overlapped candidate genes and their Jaccard similarities.

Table S3. The scale-free model fitting index R^2, the identified γ, and the degree statistical characteristics of the corresponding unweighted network for each cut-off.

Table S4. Statistical test results comparing the network features among essential and non-essential genes.

Table S5. The enrichment results of enriched Kyoto Encyclopedia of Genes and Genomes (KEGG) gene sets based on the gene set enrichment analysis (GSEA).

Table S6. The enrichment results of enriched Gene Ontology (GO) gene sets based on the gene set enrichment analysis (GSEA).

Supplementary Figures

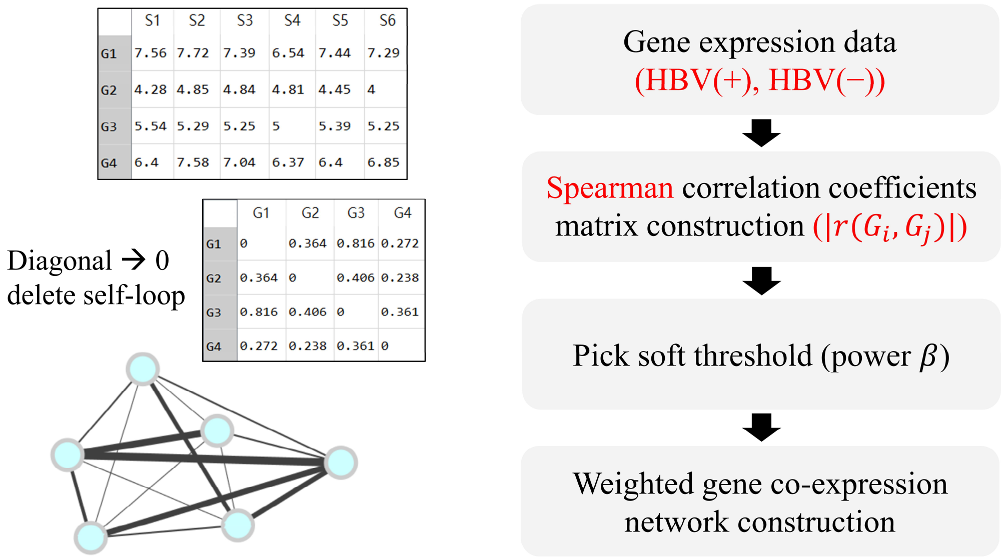

Figure S1. Steps for the construction of the weighted gene co-expression networks.

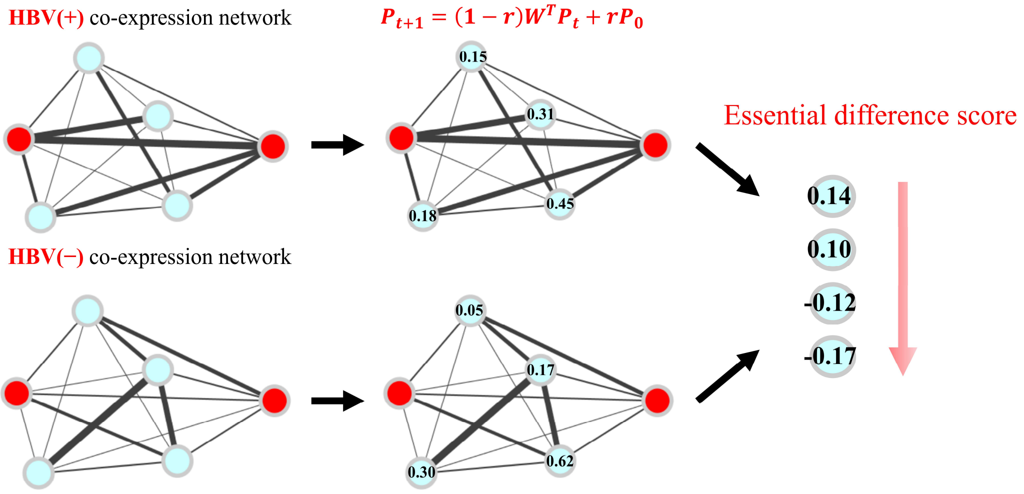

Figure S2. Steps for calculating the essential difference score using the random walk with restart (RWR) algorithm. First, 1,805 known essential genes were mapped to HBV(+) and HBV(−) co-expression networks, respectively. Second, with the 1,805 known essential genes as the seed nodes, the RWR algorithm was applied to HBV(+) and HBV(−) co-expression networks, respectively, giving essential scores for each gene in the networks. Finally, the essential difference score was calculated by subtracting the essential score of HBV(−) from the essential score of HBV(+). Genes can be ranked by sorting their corresponding essential difference score in descending order.

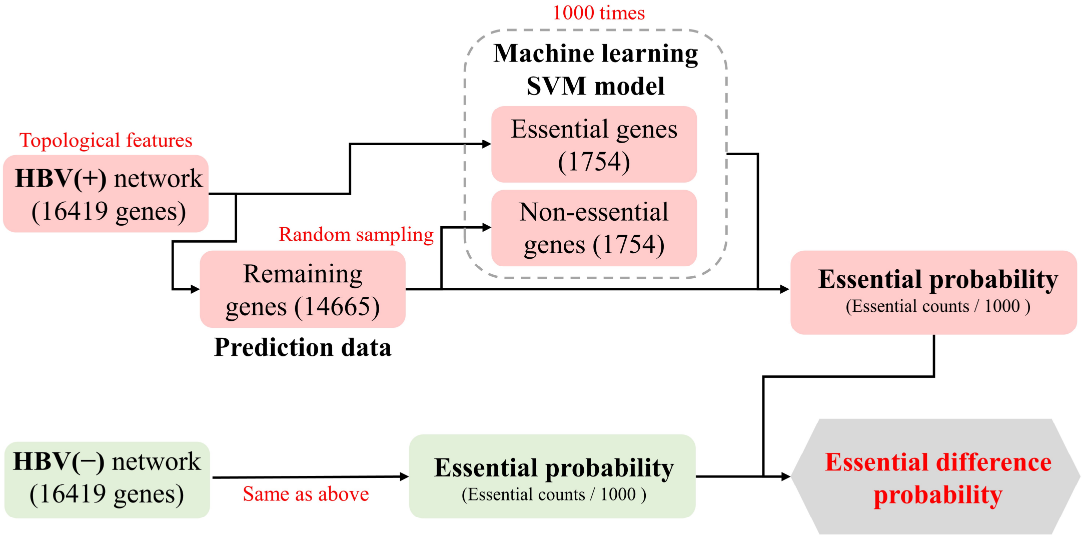

Figure S3. Steps for calculating the essential difference probability using the support vector machine (SVM) approach. First, with the network features for each gene in the networks, SVM classification models were trained to predict the CEGs. Since SVM is a supervised classification method, data with both positive and negative labels should be given for model training. In this case, the known essential genes were served as the positive group of training data. However, there are no known non-essential genes available. Therefore, the negative group of training data with the same size as positive group was randomly selected from the genes other than the known essential genes. Given the known essential genes as positive data and randomly selected genes as negative data, a SVM model can be trained to classify the remaining genes as essential or non-essential. Third, the process of random selection of negative data can be repeated 1,000 times, resulting in 1,000 SVM classification models and different number of classification results for each gene in the networks. The essential probability, which was calculated by dividing essential counts (the number of times to be predicted as essential genes) by 1,000 (the number of times as prediction data), was obtained for each gene in HBV(+) and HBV(−) networks, respectively. Finally, the essential difference probability was calculated by subtracting the essential probability of HBV(−) from the essential probability of HBV(+). Genes can be ranked by sorting their corresponding essential difference probability in descending order.

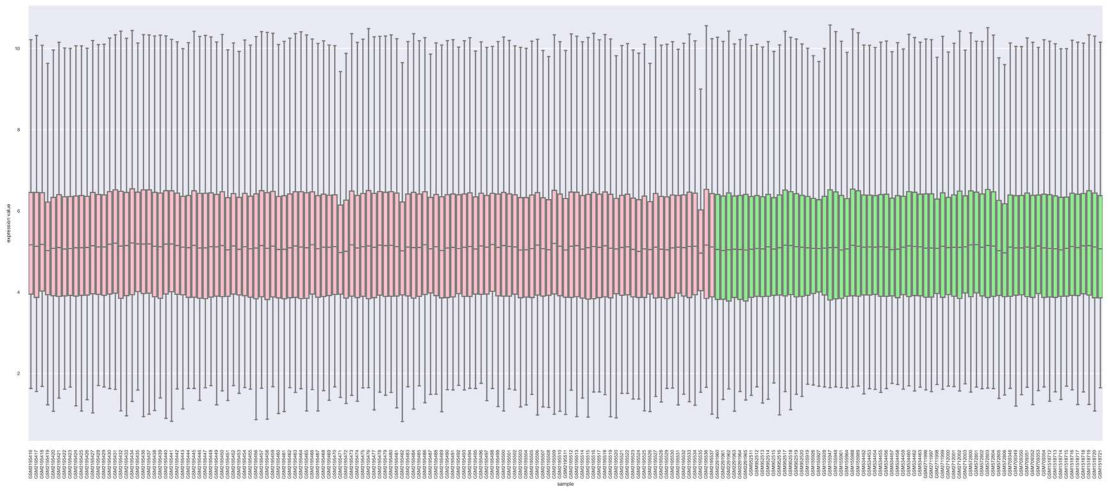

Figure S4. The gene expression profile distributions across HBV(+) and HBV(−) samples after normalization. The x-axis is the samples (pink: HBV(+) sample (n = 122), green: HBV(−) sample (n = 69)), y-axis is the gene expression value of each sample.

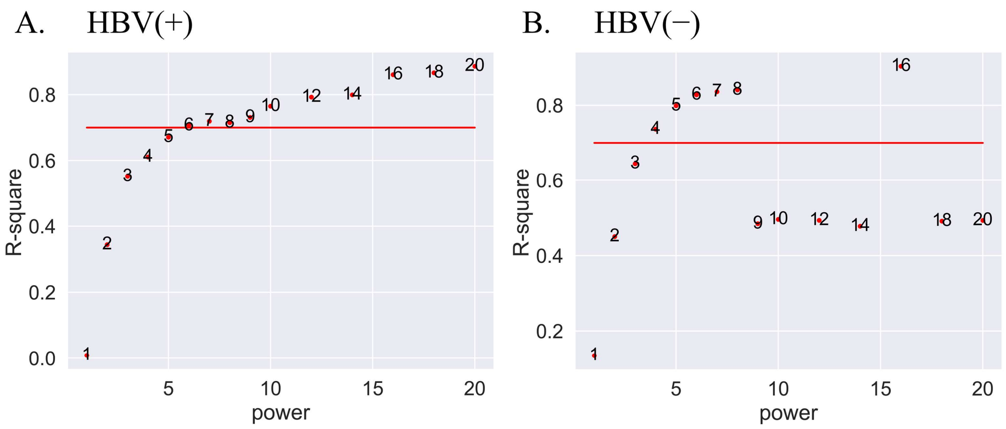

Figure S5. Different power β's and their corresponding R^2 in the scale-free topological model fitting for the soft threshold selection. (A) HBV(+). (B) HBV(−). The red line indicates that $R^2 = 0.7$.

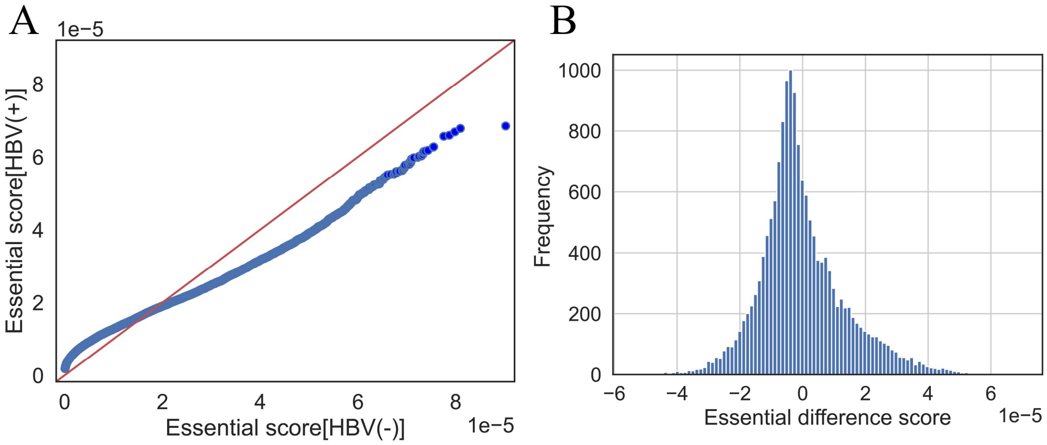

Figure S6. Distributions of the essential scores and essential difference scores. (A) Quantile-quantile plot of essential scores between genes in HBV(+) and HBV(−) networks (known essential genes excluded). (B) Distribution of essential difference scores (known essential genes excluded).

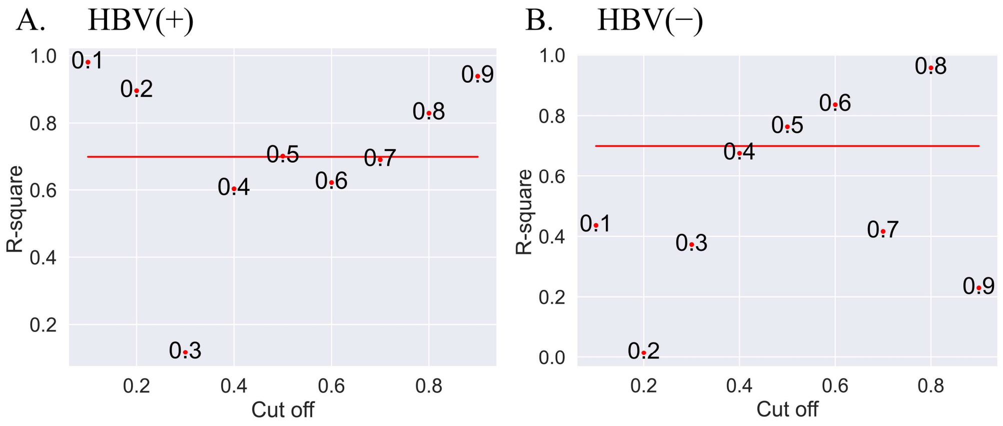

Figure S7. Different cut-offs and their corresponding R^2 in the scale-free topological model fitting for the hard threshold selection. (A) HBV(+). (B) HBV(−). The red line indicates that $R^2 = 0.7$.

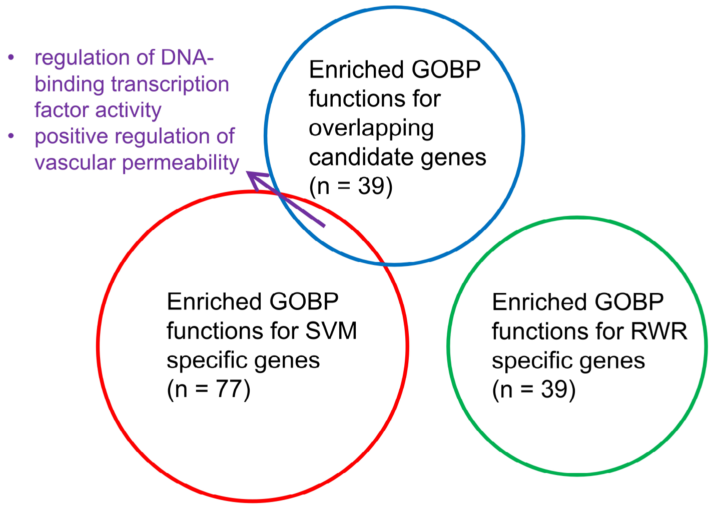

Figure S8: The Venn diagram showing the relation between the enriched GOBP functions for overlapping candidate genes, RWR-specific genes, and SVM-specific genes.

Supplementary Tables

Table S1. The number of identified candidate genes for different values of *r*.

Restart probability (*r*)	Candidate genes (n)
0.1	254
0.3	281
0.5	302
0.7	309
0.9	305

Table S2. The number of overlapped candidate genes and their Jaccard similarities.

	**0.1**	**0.3**	**0.5**	**0.7**	**0.9**
**0.1**		228 (0.74)	206 (0.59)	191 (0.51)	168 (0.43)
**0.3**	228 (0.74)		258 (0.79)	240 (0.69)	214 (0.58)
**0.5**	206 (0.59)	258 (0.79)		278 (0.83)	250 (0.7)
**0.7**	191 (0.51)	240 (0.69)	278 (0.83)		279 (0.83)
**0.9**	168 (0.43)	214 (0.58)	250 (0.7)	279 (0.83)	

The numbers in parentheses represent the Jaccard similarities of two sets of identified candidate genes.

Table S3. The scale-free model fitting index R^2, the identified γ, and the degree statistical characteristics of the corresponding unweighted network for each cut-off.

A. HBV(+)

Cut-off	R^2	γ	Mean (K)	Median (K)	Max (K)
0.1	0.980603	5.347592	12564.25	12928	15197
0.2	0.895985	1.100313	7993.673	8312	12552
0.3	0.11646	-0.18994	4531.267	4479	10053
0.4	0.6034	-0.83791	2255.267	1808	7604
0.5	0.701926	-1.24179	1001.88	520	5292
0.6	0.621923	-1.76835	396.8922	128	3236
0.7	0.691141	-2.07682	127.0204	31	1493
0.8	0.829342	-1.83637	26.36383	7	358
0.9	0.938894	-2.04021	3.582996	2	31

B. HBV(−)

Cut-off	R^2	γ	Mean (K)	Median (K)	Max (K)
0.1	0.43644	4.92443	11210.43	11311.5	14189
0.2	0.014418	0.285752	5902.369	5911	10569
0.3	0.372853	-1.30437	2518.214	2353.5	7206
0.4	0.676131	-1.85514	840.6027	648	4130
0.5	0.763495	-2.15234	212.7331	104	2039
0.6	0.836369	-2.20135	48.34284	15	799
0.7	0.41652	-3.1853	11.83646	4	236
0.8	0.958051	-2.08938	3.470305	1	47
0.9	0.229804	-3.89903	2.182648	1	12

Table S4. Statistical test results comparing the network features among essential and non-essential genes.

Feature	HBV(+)	HBV(−)
Degree (K)	998	1000
Betweenness centrality (BC)	1000	1000
Closeness centrality (CC)	1000	1000
Clustering coefficient (CCo)	1000	1000
Neighbors' intra-degree (NID)	953	1000
Essentiality index (EI)	1000	1000
Common-function degree (CFK)	1000	1000

The values in the table represent the number of significant counts among 1,000 times based on the Mann—Whitney U test.

Table S5. The enrichment results of enriched Kyoto Encyclopedia of Genes and Genomes (KEGG) gene sets based on the gene set enrichment analysis (GSEA).

Description	Size	ES	NES	p-value	adjusted p-value
Rheumatoid arthritis	74	0.38	1.88	0.000	0.009
NOD-like receptor signaling pathway	158	0.31	1.67	0.000	0.009
Osteoclast differentiation	111	0.34	1.74	0.000	0.011
Natural killer cell mediated cytotoxicity	110	0.33	1.68	0.000	0.011
Neutrophil extracellular trap formation	158	0.31	1.63	0.000	0.011
Herpes simplex virus 1 infection	394	0.25	1.42	0.000	0.021
Fc epsilon RI signaling pathway	50	0.40	1.84	0.001	0.022
Proteoglycans in cancer	170	0.29	1.56	0.001	0.022
HIF-1 signaling pathway	80	0.35	1.72	0.001	0.022
Th17 cell differentiation	94	0.33	1.66	0.001	0.030
Shigellosis	200	0.28	1.50	0.001	0.030
Yersinia infection	117	0.31	1.58	0.001	0.030
Apoptosis	107	0.32	1.64	0.001	0.032
Non-alcoholic fatty liver disease	114	0.31	1.61	0.002	0.034
Leishmaniasis	71	0.34	1.68	0.002	0.034
Toll-like receptor signaling pathway	95	0.31	1.59	0.002	0.034
EGFR tyrosine kinase inhibitor resistance	58	0.35	1.66	0.002	0.042
Epstein-Barr virus infection	161	0.28	1.48	0.003	0.045
TNF signaling pathway	104	0.30	1.54	0.003	0.047
Lipid and atherosclerosis	184	0.27	1.44	0.003	0.047
Pancreatic cancer	58	0.34	1.62	0.003	0.047
Fluid shear stress and atherosclerosis	117	0.29	1.52	0.003	0.047
Transcriptional misregulation in cancer	159	0.28	1.48	0.004	0.047
Phagosome	118	0.30	1.53	0.004	0.047
Fc gamma R-mediated phagocytosis	74	0.32	1.59	0.004	0.047
Complement and coagulation cascades	82	0.32	1.60	0.004	0.048

Table S6. The enrichment results of enriched Gene Ontology (GO) gene sets based on the gene set enrichment analysis (GSEA).

Description	Size	ES	NES	p-value	adjusted p-value	Overlapping candidate genes
myeloid leukocyte mediated immunity	429	0.28	1.63	0.000	0.000	*SERPINB6*
regulation of innate immune response	207	0.34	1.84	0.000	0.000	*CARD11* *ZBP1*
regulation of response to biotic stimulus	293	0.31	1.72	0.000	0.000	*CARD11* *ZBP1*
regulation of immune effector process	329	0.30	1.68	0.000	0.000	
leukocyte migration	374	0.29	1.64	0.000	0.000	
regulation of cytokine-mediated signaling pathway	130	0.38	1.97	0.000	0.000	*OTULIN* *PALM3* *ZBP1*
neutrophil activation	387	0.28	1.63	0.000	0.000	*SERPINB6*
RNA processing	417	0.28	1.59	0.000	0.000	*ATXN1*
granulocyte activation	393	0.28	1.61	0.000	0.000	*SERPINB6*
leukocyte degranulation	414	0.28	1.59	0.000	0.000	*SERPINB6*
antigen processing and presentation of exogenous peptide antigen via MHC class I	43	0.50	2.24	0.000	0.000	
positive regulation of leukocyte activation	263	0.30	1.64	0.000	0.001	*CARD11*
regulation of cell—cell adhesion	348	0.28	1.58	0.000	0.001	*CDH1* *CARD11*
establishment of endothelial barrier	44	0.48	2.14	0.000	0.001	
regulation of type I interferon production	85	0.40	1.97	0.000	0.001	*ZBP1*
positive regulation of immune response	476	0.25	1.47	0.000	0.002	*CARD11* *ZBP1*
regulation of cell shape	125	0.34	1.77	0.000	0.004	
JNK cascade	176	0.30	1.62	0.000	0.008	*EDA2R*
wound healing	434	0.25	1.44	0.000	0.011	*ARRB1*
positive regulation of cellular catabolic process	310	0.26	1.48	0.000	0.011	
positive regulation of protein dephosphorylation	36	0.46	1.99	0.000	0.016	
cellular response to oxidative stress	235	0.27	1.49	0.000	0.017	
glial cell activation	47	0.41	1.84	0.000	0.021	
lipoprotein catabolic process	15	0.59	2.02	0.001	0.022	
anatomical structure homeostasis	304	0.25	1.41	0.001	0.026	
coagulation	279	0.26	1.45	0.001	0.027	*ARRB1*
ribonucleoprotein complex biogenesis	125	0.31	1.62	0.001	0.028	
positive regulation of GTPase activity	334	0.25	1.41	0.001	0.028	*ARHGAP32* *ARHGEF12* *RALGAPA2*

						ARRB1
response to iron ion	28	0.47	1.92	0.001	0.031	
actin filament organization	345	0.25	1.41	0.001	0.031	*SPTBN1* *ARRB1*
cellular response to mechanical stimulus	66	0.36	1.73	0.001	0.036	
positive regulation of reactive oxygen species biosynthetic process	49	0.39	1.80	0.001	0.037	
regulation of T cell cytokine production	28	0.46	1.86	0.001	0.038	
